# Successful Surgical Treatment for Large Common Hepatic Arterial Aneurysm Incidentally Discovered during Evaluation for Mallory–Weiss Syndrome

**DOI:** 10.3400/avd.cr.25-00151

**Published:** 2026-03-03

**Authors:** Daichi Mizushima, Tsutomu Doita, Sayaka Yuzawa, Takayuki Uramoto, Naoya Kuriyama, Yuri Yoshida, Atsuhiro Koya, Mishie Tanino, Shinsuke Kikuchi, Nobuyoshi Azuma

**Affiliations:** 1 Department of Vascular Surgery, Asahikawa Medical University, Asahikawa, Hokkaido, Japan; 2 Department of Cardiovascular Surgery, The University of Osaka Graduate School of Medicine, Suita, Osaka, Japan; 3 Department of Diagnostic Pathology, Asahikawa Medical University Hospital, Asahikawa, Hokkaido, Japan

**Keywords:** common hepatic artery aneurysm, visceral artery aneurysm, Mallory–Weiss syndrome, hybrid procedure

## Abstract

Although both open and endovascular repair are available for hepatic artery aneurysms (HAAs), the optimal treatment strategy remains controversial, particularly in anatomically complex cases. In the present case, a large common HAA measuring 100 × 49 mm with a short proximal neck posed significant challenges for both modalities. To ensure intraoperative hemostasis, a hybrid approach was adopted: open surgical ligation combined with prophylactic balloon occlusion at the aneurysmal neck. This strategy enabled safe exclusion of the aneurysm without hepatic ischemia. The case underscores the importance of selecting open, endovascular, or hybrid techniques based on individual anatomical complexity to optimize outcomes in HAA management.

## Abbreviations

CHA
: common hepatic artery
HAA
: hepatic artery aneurysm
CTA
: computed tomography angiography
PHA
: proper hepatic artery
GDA
: gastroduodenal artery

## Introduction

Hepatic artery aneurysms (HAAs) are rare, accounting for approximately 20% of all visceral artery aneurysms, and are often diagnosed incidentally due to their typically asymptomatic nature. When symptoms do occur, they may include epigastric pain, gastrointestinal bleeding, and jaundice—collectively known as Quincke’s triad.^1)^ According to current Society for Vascular Surgery (SVS) guidelines, endovascular repair is generally considered the first-line treatment for HAAs owing to its minimally invasive nature and favorable perioperative outcomes.^2)^ However, in cases with complex anatomy, such as large aneurysms with short proximal necks or involvement of critical branches, open or hybrid surgical approaches may be necessary to achieve safe and effective exclusion. Herein, we report a rare case of a large common HAA (100 × 49 mm) incidentally discovered during the evaluation of Mallory–Weiss syndrome, which was successfully treated with a hybrid procedure combining open ligation and prophylactic balloon occlusion.

## Case Report

**Fig. 1:**
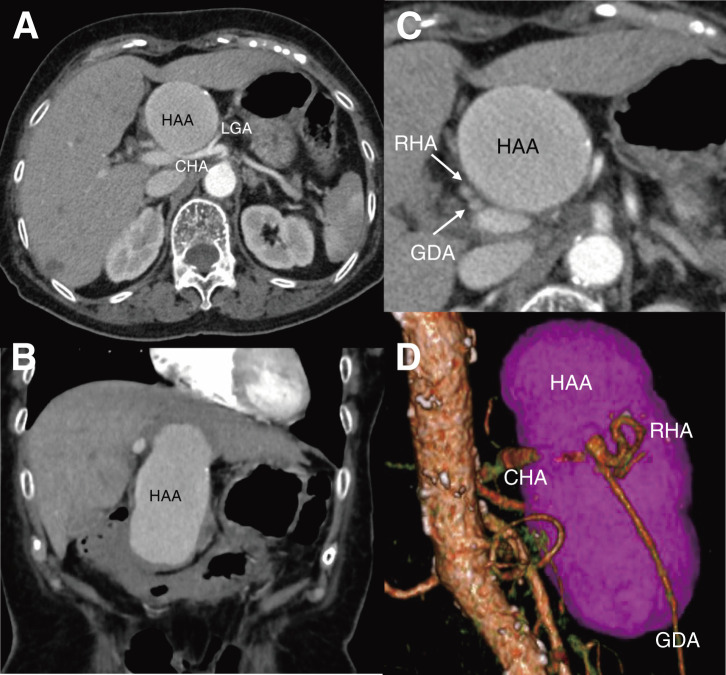
Preoperative computed tomography angiography demonstrating a large common HAA measuring 100 × 49 mm. (**A**) Axial view and (**B**) coronal view showing the aneurysm compressing the gastric antrum. (**C**) The aneurysm is located proximal to the GDA and RHA. (**D**) Three-dimensional posterior view showing a short distal landing zone from the bifurcation of the GDA and proper hepatic artery. CHA: common hepatic artery; HAA: hepatic artery aneurysm; LGA: left gastric artery; RHA: right hepatic artery; GDA: gastroduodenal artery

An 80-year-old female with a history of hypertension, hyperuricemia, and total hysterectomy for uterine fibroids 33 years earlier, and no history of alcohol abuse, was referred to the previous hospital for hematemesis. She had experienced epigastric pain for 2 weeks and had been treated with oral proton pump inhibitors by her primary physician. An upper gastrointestinal endoscopy performed at that time revealed no abnormalities. However, an emergent endoscopy at the referring hospital demonstrated mucosal lacerations at the 5 o’clock and 7 o’clock positions of the esophagogastric junction, with active bleeding from the former site, which was successfully managed with endoscopic clipping. She was diagnosed with Mallory–Weiss syndrome. Subsequent computed tomography angiography (CTA) incidentally revealed a large common HAA measuring 100 × 49 mm (**Fig. 1A** and **1B**), without involvement of other visceral arteries. She was hemodynamically stable upon transfer to our department for further management. However, due to the aneurysm’s massive size, precise evaluation of the proximal and distal landing zones was limited on imaging (**Fig. 1C** and **1D**). The uncertain proximal neck anatomy, short distal landing zone beyond the gastroduodenal artery (GDA), and compression of adjacent organs rendered endovascular exclusion technically challenging and unlikely to provide sufficient symptomatic relief. The proximal common hepatic artery (CHA) measured approximately 7.5 mm in diameter, while the distal CHA was about 4.5 mm, resulting in a diameter mismatch that further complicated the feasibility of endovascular repair. Therefore, semi-urgent open surgical repair was planned. Given the potential risk of aneurysmal rupture during dissection and the anticipated difficulty in achieving rapid hemostatic control, a hybrid approach was selected. A balloon catheter was preemptively positioned at the proximal neck of the aneurysm to allow immediate occlusion in the event of intraoperative bleeding. Furthermore, because preoperative imaging provided limited hemodynamic information due to the aneurysm’s massive size, intraoperative angiography was planned to assess hepatic arterial flow and guide surgical decision-making. This strategy enabled both real-time vascular assessment and secure bleeding control, facilitating a safe and effective open repair.

**Fig. 2:**
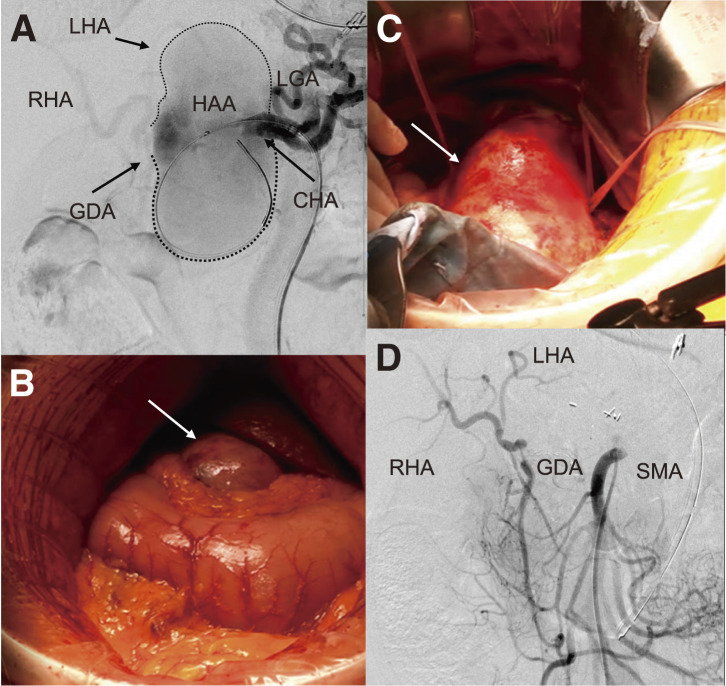
Intraoperative findings. (**A**) Angiography from the celiac artery before surgical exposure. An 8 × 40-mm balloon catheter is placed in the CHA and a common HAA for proximal inflow control. A short distal neck of the CHA is identified, along with the GDA and both hepatic arteries. The aneurysm is outlined by dotted lines. (**B**) The aneurysm is exposed within the lesser omentum. (**C**) The proximal and distal CHA are taped following dissection. (**D**) Angiography from the SMA after CHA ligation confirms retrograde hepatic perfusion via the GDA. CHA: common hepatic artery; HAA: hepatic artery aneurysm; LGA: left gastric artery; RHA: right hepatic artery; LHA: left hepatic artery; GDA: gastroduodenal artery; SMA: superior mesenteric artery

Under general anesthesia, the right common femoral artery was accessed, and selective catheterization of the celiac artery was performed. A 0.035-inch Radifocus guidewire (Terumo, Tokyo, Japan) was advanced, over which a Parent Plus 45 guiding sheath (Medikit, Tokyo, Japan) was positioned within the celiac artery. To allow immediate proximal control and reduce the risk of intraoperative aneurysmal rupture during dissection, an 8 × 40 mm Sterling balloon catheter (Boston Scientific, St. Paul, MN, USA) was preemptively placed from the celiac artery into the CHA. Intraoperative angiography revealed that the distal neck was too short to ensure secure landing for endovascular exclusion. Based on this finding, open surgical ligation was deemed necessary to achieve definitive aneurysm control (**Fig. 2A**). Following an upper midline laparotomy, the aneurysm was identified within the lesser omentum. After incising the lesser omentum, the anterior wall of the aneurysm was exposed (**Fig. 2B** and **2C**). Dissection was initiated from the left side, allowing identification of the proximal CHA and the splenic artery. Subsequent dissection from the right side revealed the distal CHA and the GDA. After systemic heparinization, the CHA was clamped proximally and distally to the aneurysm. The aneurysm sac was opened, and the proximal CHA was closed using a continuous 5-0 polypropylene suture. Despite proximal clamping, Doppler ultrasonography confirmed preserved pulsatile flow in the proper hepatic artery (PHA). The distal CHA was then ligated with double 1-0 silk ties. No communication was observed between the aneurysm and the gastrointestinal tract; therefore, partial resection and plication of the aneurysmal wall were performed. Intraoperative angiography via the superior mesenteric artery demonstrated adequate retrograde perfusion of the PHA through the GDA (**Fig. 2D**).

**Fig. 3:**
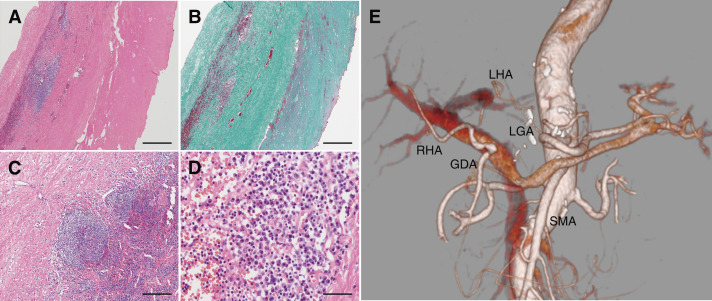
Histopathological findings of the aneurysmal wall and postoperative CTA. (**A**) H&E staining reveals a pseudoaneurysm with lymphoplasmacytic infiltration. The lumen is located on the right side and the adventitia on the left. (**B**) Elastica–Masson staining shows diffuse fibrosis and complete loss of the internal and external elastic laminae and medial smooth muscle cells, with organizing thrombi adherent to the lumen. (**C**) Lymphoid follicles are present focally. (**D**) Dense infiltration of plasma cells and lymphocytes is observed. Scale bars: (**A**, **B**) 500 μm, (**C**) 200 μm, and (**D**) 50 μm. Postoperative CTA demonstrates good perfusion of both hepatic arteries from the SMA via the GDA (**E**). CTA: computed tomography angiography; H&E: hematoxylin and eosin; LGA: left gastric artery; RHA: right hepatic artery; LHA: left hepatic artery; GDA: gastroduodenal artery; SMA: superior mesenteric artery

Histopathological examination revealed a pseudoaneurysm with lymphoplasmacytic infiltration. The aneurysmal wall consisted of fibrous tissue, lacking both internal and external elastic laminae and medial smooth muscle cells. Organizing thrombi adhered to the lumen, and lymphoplasmacytic infiltrate with focal lymphoid follicles was observed on the adventitial side of the aneurysmal wall. Few neutrophils were present, but no bacteria or fungi were identified by Gram and Grocott staining. Immunohistochemically, the lymphocytes comprised CD3-positive T cells and CD20-positive B cells, with many plasma cells highlighted by an anti-CD138 antibody. No light-chain restriction was observed. Although there were 20 IgG4-positive cells/mm^2^, the IgG4/IgG ratio was about 10%. Immunostaining for *Treponema* was negative. No granulomas and multinucleated giant cells were seen (**Fig. 3A**–**3D**). These pathological findings are consistent with an inflammatory pseudoaneurysm, in the absence of evidence for specific vasculitis or infection. She was discharged on postoperative day 14. Postoperative CTA showed preservation of hepatic arterial perfusion from the SMA through the GDA (**Fig. 3E**). Despite inflammatory pathological features, there was no evidence of systemic vasculitis or multisite vascular involvement. Thus, additional medical therapy such as corticosteroids was not considered necessary. Given the inflammatory nature of the pseudoaneurysm, annual CTA follow-up was performed to ensure no recurrence at the treated site and no aneurysmal changes in other vessels. Her postoperative course was uneventful for 5 years. No elevation of her liver enzymes was observed and her abdominal pain resolved after surgery.

## Discussion

HAAs are often asymptomatic and discovered incidentally; however, when symptoms occur, they may reflect mass effect or rupture. In our case, the patient presented with epigastric pain and hematemesis, ultimately diagnosed as Mallory–Weiss syndrome. The aneurysm was located adjacent to and compressing the gastric antrum, which may have contributed to repeated vomiting and mucosal laceration at the esophagogastric junction. Although gastrointestinal bleeding is a recognized manifestation of symptomatic HAAs, most reported cases involve direct fistulization between the aneurysm and the gastrointestinal tract.^3)^ In contrast, our case is rare in that the bleeding was possibly secondary to mechanical compression of the gastric antrum by the aneurysm, rather than due to aneurysmal rupture or fistula formation. Although a direct causal relationship cannot be definitively established, the anatomical proximity and clinical presentation suggest that the aneurysm may have contributed to the development of Mallory–Weiss syndrome.

In symptomatic HAAs with significant mass effect, endovascular repair may reduce aneurysmal pressure but may not adequately relieve symptoms caused by organ compression.^4)^ Therefore, when feasible, open surgical repair should be considered, especially in cases where symptom resolution is a primary therapeutic goal. In anatomically complex cases, a hybrid approach incorporating intraoperative angiography and endovascular control can enhance procedural safety and facilitate tailored surgical decision-making. In the present case, this strategy proved particularly valuable. The endovascular component provided a reliable means of proximal control, offering a safeguard against potential injury or rupture during dissection of the large aneurysm. Moreover, given the technical difficulty of endovascular exclusion due to the short distal neck and uncertain proximal anatomy, open repair was deemed more appropriate. In addition, intraoperative angiography allowed real-time assessment of hepatic arterial perfusion via the GDA following CHA clamping, confirming adequate collateral flow from the SMA. Thus, the hybrid approach not only ensured intraoperative safety but also provided critical hemodynamic information that guided definitive surgical management.

As for treatment, current guidelines recommend intervention for all symptomatic HAAs and favor open repair for aneurysms larger than 5.0 cm in diameter.^2)^ Both open and endovascular approaches are available, but the optimal modality remains controversial and should be determined based on clinical presentation, aneurysm type and location, and patient-specific risk factors.^5)^ When hepatic perfusion can be maintained via collateral flow from the GDA and right gastric branches, either open ligation or endovascular embolization may be considered.^2)^ Importantly, large aneurysm size alone is not a contraindication for endovascular treatment, and favorable outcomes have been reported even in cases of giant HAAs.^6,7)^ In the present case, however, the aneurysm was very large, symptomatic, and located directly on the CHA, without involvement of other visceral branches. Open surgical ligation was considered appropriate given the aneurysm’s size and the preserved hepatic perfusion following CHA exclusion. Although CHA reconstruction was considered, it was not pursued because adequate hepatic arterial perfusion was maintained via collateral flow through the GDA, as confirmed intraoperatively by Doppler ultrasonography. In addition, intraoperative angiography demonstrated satisfactory opacification of the PHA and hepatic arteries. This strategy is consistent with established surgical principles in pancreaticoduodenectomy, where arterial reconstruction is generally not required if hepatic arterial pulsation and biphasic Doppler waveforms are preserved during the GDA clamp test.^8)^ Despite the inability to objectively quantify the adequacy of PHA inflow from the GDA, the absence of any hepatic dysfunction during the 5-year postoperative course supports the appropriateness of our intraoperative hemodynamic assessment.

Although endovascular exclusion might have been technically feasible by placing a stent graft with its distal landing zone in the PHA or GDA, significant luminal diameter mismatch between the CHA and these distal vessels, along with concerns regarding hepatic perfusion, would have posed a high risk of treatment failure. In such scenarios, revascularization using a prosthetic or vein graft may be required to prevent hepatic ischemia, particularly when the aneurysm involves the GDA or PHA. Therefore, accurate preoperative assessment of aneurysm location and vascular anatomy using CT or angiography is essential for selecting an appropriate treatment strategy and ensuring hepatic arterial integrity.

## Conclusion

Hybrid treatment is effective for managing common HAAs, especially in symptomatic or complex cases. It enables real-time assessment of hepatic flow and provides proximal control, allowing prompt response to aneurysmal injury or rupture. Careful preoperative imaging is essential for optimal strategy selection.
